# Effects of different processing methods on the antioxidant and immune stimulating abilities of garlic

**DOI:** 10.1002/fsn3.942

**Published:** 2019-02-28

**Authors:** Eric Banan‐Mwine Daliri, Se‐Hun Kim, Byun‐Jae Park, Hee‐Sung Kim, Jung‐Mi Kim, Hyong Seo Kim, Deog‐Hwan Oh

**Affiliations:** ^1^ Department of Food Science and Biotechnology Kangwon National University Chuncheon South Korea; ^2^ Daoom Company Sacheon City, Gyeongsangnam‐do South Korea

**Keywords:** fermentation, garlic, oxidative stress, proinflamatory cytokines, solid‐state fermentation

## Abstract

In this study, we determined the antioxidant and immune stimulating abilities of a garlic product developed by freeze drying, heat drying, and solid‐state fermentation of heat‐dried garlic. *Lactobacillus plantarum* KCTC21004 and *Leuconostoc mesenteroides* KCTC13302 were used for the sample fermentation. The optimum conditions for fermentation were 50% (v/w) moisture, a fermentation time of 48 hr and a temperature of 37°C. Heat‐dried garlic samples fermented with *L. plantarum* KCTC21004 (HD21004) and *L. mesenteroides* KCTC13302 (HD13302) showed the highest flavonoid contents while heat‐dried garlic (HD) had the lowest flavonoid content. HD21004 contained the highest phenolic compounds, showed the highest antioxidant activity and demonstrated a strong immune stimulating effect while freeze‐dried garlic showed the lowest flavonoid and polyphenolic contents. Overall, the heat‐dried garlic samples (fermented and unfermented) contained about three times more *S*‐Allylcysteine (SAC) than the freeze‐dried samples (FD). The current study demonstrates that heat drying and subsequent fermentation of garlic with *L. plantarum* KCTC21004 can improve its therapeutic effects.

## 1 INTRODUCTION

Oxidative stress can cause cell death (Nakajima et al., 2017) and can promote the activation of mediator signaling molecules such as NF‐kB to increase inflammatory cytokine production (Buelna‐Chontal & Zazueta, 2013). However, endogenous antioxidants such as Glutathione play critical roles in defending against oxidative stress to protect host immune cells from free radicals. Therefore, daily consumption of functional foods that have antioxidant activities could be a good method of mitigating chronic inflammation and boosting the immune system (Arranz, Fernández, Rodríguez, Ribera, & De Fuente, 2008).Hence for centuries, garlic (*Allium sativum* L.) has been used in medicine, condiments, seasonings, and health foods all over the world. Recent studies have reported many beneficial functions of garlic including antimicrobial effect (Hosseini, Bayat, Shabani, Mozaffari, & Amir, 2014), anticancer effect (Zong & Martirosyan, 2018), as well as cholesterol‐lowering effect (Ried, 2016). Although garlic has many active components that contribute to its health benefits, consumption of unprocessed raw garlic is limited due to its characteristic odor, taste, and tendency to cause stomach upset. Therefore, in recent years, various processing methods such as heat treatment (Wang, Zhang, & Jing, 2016), aging and fermentation (Kim et al., 2016) have been used to eliminate the unpleasant odor and improve the palatability of garlic products. Several methods such as freeze drying and heating have been applied to solve this problem. However, the most widely used processing method for removing the unpleasant odor and taste of garlic is heat treatment (García‐Villalón et al., 2016). During heat treatment, various physicochemical changes occur, including changes in odor, nutrient content, flavor and color.An important compound, *S*‐Allylcysteine, is produced in large amounts during the aging process of garlic and is a key compound responsible for the multiple pharmacological activities of garlic such as antioxidant, anticancer, and neurotrophic activities (Baluchnejadmojarad, Kiasalari, Afshin‐Majd, Ghasemi, & Roghani, 2017; Ho et al., 2018). *S*‐Allylcysteine is formed by the enzymatic hydrolysis of γ‐glutamyl‐*S*‐allyl cysteine (GSAC) by γ‐glutamyl transpeptidase (γ‐GTP) (Kodera et al., 2002). The activity of γ‐GTP is influenced by temperature and the levels of SAC tend to increase when garlic is heated (Bae, Cho, Won, Lee, & Park, 2014). For this reason, though raw garlic contains 20–30 μg/g of SAC (Bae et al., 2014), the amount of SAC in black garlic (a heat‐treated garlic product) is five to six times higher than that in raw garlic (Wang et al., 2010). There is however the tendency of heat treatment to destroy heat sensitive yet bioactive compounds in garlic. Milder techniques such as freeze drying could be a good alternative. However, the effects of this process on bioactivity of the final product remain unknown. Other studies have shown that fermenting garlic with lactic acid bacteria (LAB) significantly improves its pharmacological effects (Lee, Cho, Kim, & Moon, 2016; Lee, Joo, & Kwon, 2016; Lee, Lee, Yu, Lee, & Cho, 2017). By submerged fermentation, garlic has been fermented with Pediococcus (Ham et al., 2010) and Monascus species (Sumioka, Hayama, Shimokawa, Shiraishi, & Tokunaga, 2006) in an effort to improve its antimicrobial and hypolipidemic activities. Nonetheless, the garlic‐derived bioactive compounds contained in these fermented products remain unknown. Also, very few reports are available on the effects of heat drying and subsequent solid‐state fermentation on the biological activities of garlic (Lee, Cho, Kim, & Moon, 2016; Lee, Joo, & Kwon, 2016).

Therefore, in this study, we investigated the effects of freeze drying, heat drying, and solid‐state fermentation on the antioxidant and immune stimulating effects of garlic.

## 2 MATERIALS AND METHODS

### 2.1 Bacteria strains and growth conditions

Three lactic acid bacteria strains namely *Lactobacillus plantarum* KCTC21004 and *Leuconostoc mesenteroides* KCTC13302 were obtained from the Department of Food Science and Biotechnology, Kangwon National University, Korea. These strains were chosen for the fermentation process because they yielded biologically potent fermented products in our early study (data not shown). The bacteria were cultured in de Man, Rogosa and Sharpe broth (MRS, MBCell‐ Korea) at 37°C overnight. The culture was centrifuged at 5,000×g for 10 min at room temperature, and the pellets were washed twice with distilled water.

### 2.2 Solid‐state fermentation and optimization

Heat‐dried garlic powder (HD) was produced by hot air oven drying of fresh garlic slices at 60°C for 48 hr. On the other hand, freeze‐dried garlic (FD) was produced by freezing fresh garlic slices at −80°C for 8 hr and keeping in a TFD5505 table top freeze dryer (ilshinBioBase Co. Ltd, South Korea) for 48 hr. The dried garlic samples were ground into powder using a Philips HL 1645 grinder (Koninklijke Philips, India), and the moisture was adjusted.

To ascertain the optimum moisture content required for the fermentation process, flasks containing 10 g of heat‐dried garlic powder mixed with varying amounts of water (20%, 30% 40% and 50% v/w) were plugged with nonadsorbent cotton and autoclaved at 121°C for 15 min. Each sample was inoculated with 10^6^ cfu/ml of the bacteria culture and incubated at 37°C. The total colony forming units of the bacteria in the samples were enumerated after 24, 48, and 72 hr on MRS agar plates. The minimum moisture content and fermentation time that enhanced high cell number was chosen as the optimum condition for the fermentation process.

The samples were freeze‐dried and extracted with 70% ethanol (v/v), and the extracts were used for further studies.

### 2.3 Analysis of bioactive compounds

#### 2.3.1 Analysis of flavonoids

In brief, 50 μl of the samples (1 mg/ml ethanol) were made up to 1 ml with methanol, mixed with 4 ml of distilled water and then 0.3 ml of 5% NaNO_2_ solution; 0.3 ml of 10% AlCl_3_ solution was added after 5 min of incubation, and the mixture was allowed to stand for 6 min. Then, 2 ml of 1 M NaOH solution was added, and the final volume of the mixture was brought to 10 ml with double‐distilled water. The mixture was allowed to stand for 15 min, and absorbance was measured at 510 nm with a biospectrometer (Eppendorf Biospectrometer^®^ fluorescence, Eppendorf Korea Ltd., Korea**)**. The total flavonoid content was calculated from a calibration curve, and the result was expressed as milligram quercetin equivalent (QE) per gram dry weight.

#### 2.3.2 Analysis of phenolic compounds

The total phenolic content was determined according to the method of Koley, Kaur, Nagal, Walia, and Jaggi (2016) with slight modifications. Samples of the extracts (200 mg) were dissolved in 1 ml of distilled water and filtered and 100 μl was oxidized with 2.5 ml of 10% Folin–Ciocalteau's reagent (v/v) inside a test tube. The samples were then neutralized by adding 2.0 ml of 7.5% sodium carbonate. The reaction mixture was incubated for 40 min at 45°C, and the absorbance was measured at 765 nm with a biospectrometer. The total phenolic content of the samples was subsequently calculated from a standard curve of absorbance of gallic acid and reported as gallic acid equivalent (GAE).

#### 2.3.3 S‐Allyl cysteine analysis

S‐Allyl cysteine (SAC) was analyzed by high‐performance liquid chromatography (HPLC; Waters Co., Milford, MA, USA) as described previously (Lee et al., 2017). SAC was analyzed by derivatization with fluorescent probes (Waters AccQ‐Tag chemistry package, Waters Co.). HPLC conditions were as follows: the column used was AccQ‐Tag (3.9 × 150 mm, 4 μm; Sigma Chemical Co., St. Louis, MO, USA), the column temperature was 37°C, the flow rate was 1.0 ml/min, the mobile phase was AccQ‐Tag Eluent A and 60% acetonitrile under gradient conditions (100:0 to 0:100, v/v), the wavelength was 254 nm, and the injection volume was 10 μl.

### 2.4 Antioxidant activity

#### 2.4.1 DPPH radical scavenging activity

The free radical scavenging activity of all the extracts was evaluated using 1, 1‐diphenyl‐2‐picryl‐hydrazyl (DPPH) according to the previously reported method by Shen et al. (2010). Briefly, 3.9 mg of DPPH was dissolved in 100 ml of methanol to obtain 0.1 mM DPPH solution. An aliquot (1 ml) of DPPH solution was added to 3 ml of garlic extracts dissolved in methanol (50, 100, 200, 400, and 800 μg/ml). The mixture was vigorously shaken and allowed to stand at room temperature for 30 min. Absorbance was measured at 517 nm using a spectrophotometer. Lower absorbance values of reaction mixture indicated higher free radical scavenging activity.

The capability of scavenging the DPPH radical was calculated as:

$$\text{DPPH scavenging effect}\;\left(\%\text{inhibition}\right)=(\left(A_{0}-A_{1}\right)/A_{0})\times 100)$$

where, *A*_0_ is the absorbance of the control reaction, and *A*_1_ is the absorbance in the presence of all of the extract samples and reference. All the tests were performed in triplicates and the results were averaged (Figure 4).

#### 2.4.2 ABTS radical scavenging activity

##### Preparation of ABTS radical cation stock solution

ABTS stock solution was prepared according to the manufacturer's instructions (Sigma‐Aldrich, Korea). ABTS solution was diluted with methanol to obtain an absorbance of 0.70 at 734 nm. Serial concentrations of garlic extracts (50, 100, 200, 400, and 800 μg/ml) were prepared in methanol. An aliquot of the garlic extracts (1 ml) was added to 2.5 ml of the ABTS solution, and the absorbance at 734 nm was read after 7 min using the spectrophotometer.

The percentage inhibition was calculated as:

$$\text{ABTS radical scavenging activity}\;\left(\%\right)=\left(A_{\mathrm{control}}-A_{\mathrm{extracts}}\right)/A_{\mathrm{control}}\times 100$$

### 2.5 Immune stimulation ability

#### 2.5.1 Peripheral blood mononuclear cells (PBMC) culture

The PBMCs were isolated from 5 ml of whole blood consisting of anticoagulant EDTA (Sigma‐Aldrich) from a healthy adult donor on Ficoll‐Hypaque (Hornby, Ontario, Canada) by centrifugation at 400×g at room temperature for 30 min according to the manufacturers’ instructions. The cells were cultured in T25 culture flask (SPL Life Sciences, Pocheon, Korea) overnight in RPMI‐1640 media (Sigma‐Aldrich) supplemented with fetal bovine serum (FBS), 100 U/ml penicillin, 100 μg/ml streptomycin (Sigma‐Aldrich) and 2 mM L‐glutamine (Gibco, NY, USA) at 37°C for 24 hr before any treatments. Before carrying out the experiment, the medium was discarded; the separated cells were washed and counted. The cell viability was measured by trypan blue staining to >95% viability.

#### 2.5.2 Cell proliferation enhancement ability

Cell proliferation was determined by MTS colorimetric assay as described before (Zhang et al., 2017). Peripheral blood mononuclear cells (6 × 10^3^ cells/well) were seeded in 96‐well culture plates. After treating the cells with 750 μg/ml of each extract, 10 μl of MTS solution (MTS Assay Kit ab197010, Dawinbio Inc., Seoul‐Korea) was transferred to each well containing 100 μl of medium and incubated at 37°C for 4 hr in accordance with the manufacturer's instructions. Nontreated cells were used as control. Colorimetric analysis was determined using an ELISA plate reader (DTX880; Beckman, Miami, FL, USA) at 500 nm.

#### 2.5.3 Cytokine assay

A TNF‐α ELISA kit (eBioscience, Vienna, Austria) was used for measuring the TNF‐α levels according to the manufacturer's instructions. Briefly, cytokine measurement was performed on the supernatant of the cultured PBMC after they were treated with 750 μg/ml of each ethanolic extract and incubated for 72 hr. Supernatants from untreated cells were used as control. The absorbance of the supernatants was measured at 450 nm. The concentrations of TNF‐α were calculated by converting the absorbance values, using a standard curve prepared with serial dilutions of the recombinant TNF‐α standards.

#### 2.5.4 Determination of nitric oxide (NO) production

Briefly, PBMC (99 μl, plated at 10^6^ cells/ml) were treated with extracts (1 μl) and incubated for 72 hr. Nitrite was measured in the supernatant using the Griess reaction. Supernatants from untreated cells were used as control. The culture media of the PBMC (80 μl) were mixed with 80 μl of Griess reagent (Sigma‐Aldrich), and its absorbance was measured at 550 nm using an Eppendorf biospectrometer. The nitrite concentrations in the culture media were determined by comparing them with a NaNO_2_ standard curve. Each concentration was assayed three times.

### 2.6 Statistical analysis

All experiments were carried out in triplicates and the results were expressed as the mean ± standard deviation. The statistical analysis of data was performed using GraphPad Prism 5.0 (2007) statistical software system (GraphPad Software Inc., CA, USA). *p *< 0.05 was considered significant.

## 3 RESULTS AND DISCUSSION

Garlic is known for its health effects, yet processing may affect their therapeutic effects. However, though heating destroys certain active ingredients such as allicin (an antimicrobial compound), the levels of other bioactive compounds including SAC increase significantly.

### 3.1 Solid‐state fermentation and optimization

**Figure 1:**
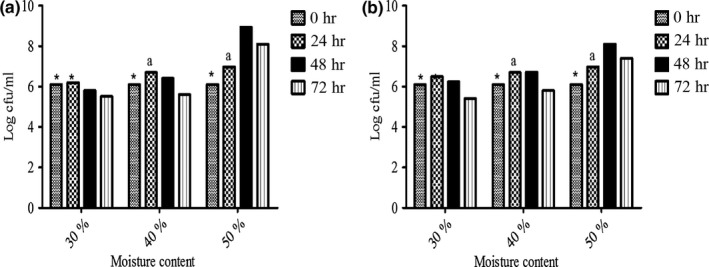
Viability of (a) *Lactobacillus plantarum* KCTC21004 and (b) *Leuconostoc mesenteroides* KCTC13302 in heat‐dried garlic with varying moisture content and fermentation time

Microorganisms require a threshold of moisture for growth. At a moisture content of 50% (v/w), the bacteria population of all the strains increased significantly relative to the lower moisture levels. For this reason, the optimum moisture content required for the fermentation process was set at 50% (v/w). After 48 hr of fermentation at 37°C (at 50% moisture content), the LAB population was over 8 Log cfu/ml (Figure 1).

### 3.2 Antioxidant activities

**Figure 2:**
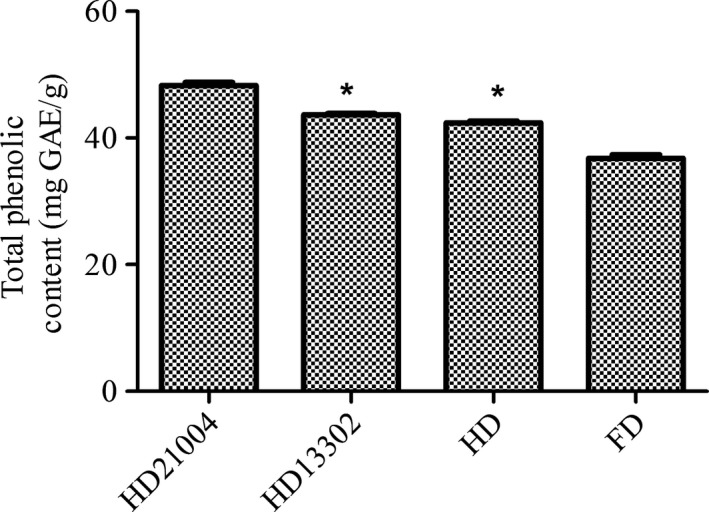
Total phenolic content in heat‐dried garlic fermented with KCTC21004 (HD21004), heat‐dried garlic fermented with KCTC13302 (HD13302), heat‐dried garlic (HD), and freeze‐dried garlic (FD)

**Figure 3:**
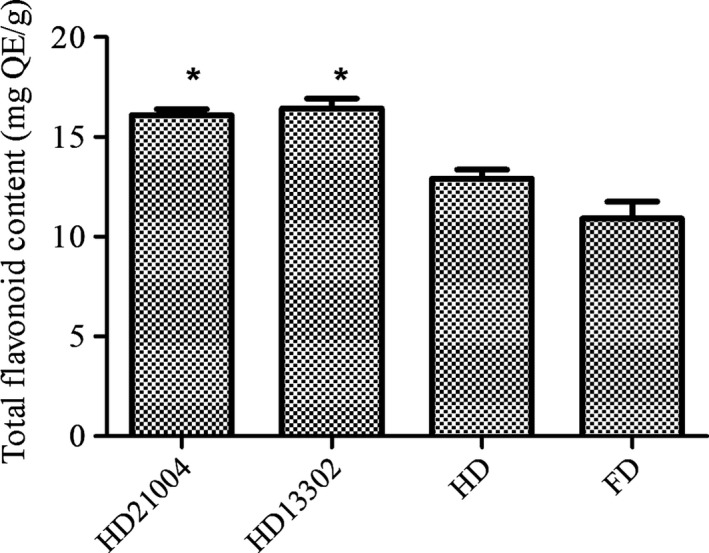
Total flavonoid content in heat‐dried garlic fermented with KCTC21004 (HD21004), heat‐dried garlic fermented with KCTC13302 (HD13302), heat‐dried garlic (HD), and freeze‐dried garlic (FD)

Phenolic and flavonoid compounds are very important constituents of plants because as they act as free radical scavengers. Although samples fermented with *L. plantarum* KCTC21004 recorded the highest level of total phenolic compounds (48.28 ± 0.5 mg GAF/g), the total phenolic contents in samples fermented with *L. mesenteroides* KCTC13302 and heat‐dried unfermented garlic were not significantly different. Meanwhile, freeze‐dried garlic showed the least total phenolic contents (Figure 2). *Lactobacillus plantarum* and *L. mesenteroides* fermented samples, however, showed similar flavonoid contents which were higher than heat‐dried garlic. The freeze‐dried samples, however, showed the least flavonoid contents (Figure 3). This observation is in accordance with earlier reports that showed that fermented vegetables have higher phenolic and flavonoid compounds than their unfermented counterparts (Adetuyi & Ibrahim, 2014). Naturally, phenolic compounds are bound to sugar molecules which reduce their bioavailability after consumption. During fermentation however, proteolytic enzymes from microorganisms hydrolyze complexes of polyphenol into free and soluble phenols which are more active and readily absorbed (Ademiluyi & Oboh, 2011; Shrestha, Dahal, & Ndungutse, 2010). Other studies have also reported that LAB release β‐glucosidase which hydrolyze flavonoid‐bound compounds to make flavonoids readily available (Lee, Cho, Kim, & Moon, 2016; Lee, Joo, & Kwon, 2016; Yang et al., 2012). Our results therefore demonstrate that the ability of such bioactive molecules to be released during fermentation is strain dependent.

**Figure 4:**
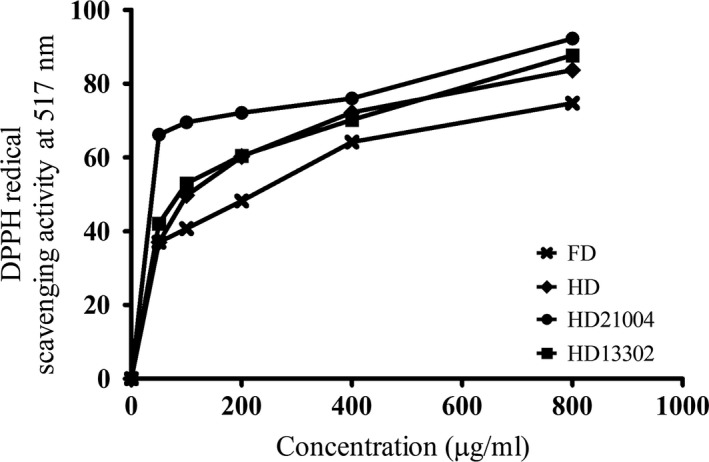
DPPH radical scavenging activities of garlic processed by different methods

**Figure 5:**
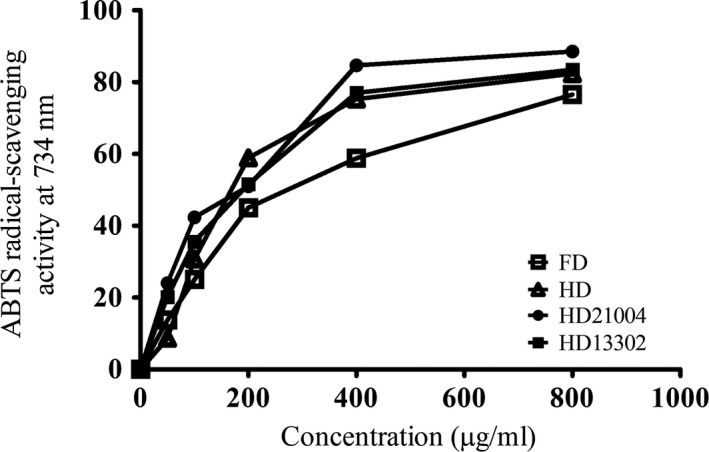
ABTS radical scavenging activities of garlic processed by different methods

The antioxidant effect was evaluated using two widely used methods; DPPH assay and ABTS assay. The DPPH radical has a characteristic absorption at 517 nm in ethanol, which disappears with the acceptance of an electron from antioxidant molecules (Lee, Cho, Kim, & Moon, 2016; Lee, Joo, & Kwon, 2016). All the fermented samples showed better DPPH radical scavenging activity than the unfermented samples. *Lactobacillus plantarum* fermented samples, however, showed the strongest DPPH radical scavenging activity and the activity increased with increasing concentration (Figure 4). Similarly, the fermented samples showed better ABTS radical scavenging activities (Figure 5) in the same order observed in the DPPH assay (HD21004 > HD13302 > HD > FD). This observation agrees with earlier studies that reported that the phenolic contents of plant materials are directly related to their antioxidant abilities (Demir, Yildiz, Alpaslan, & Hayaloglu, 2014; Zhang et al., 2014). During heat treatment, nonenzymatic browning reactions such as the Maillard reaction, caramelization and chemical oxidation of phenols occur. Such nonenzymatic browning reactions may result in the formation of compounds with strong antioxidant properties and this might have contributed to the strong antioxidant effects of the heated samples.

### 3.3 Immune stimulating ability

**Figure 6:**
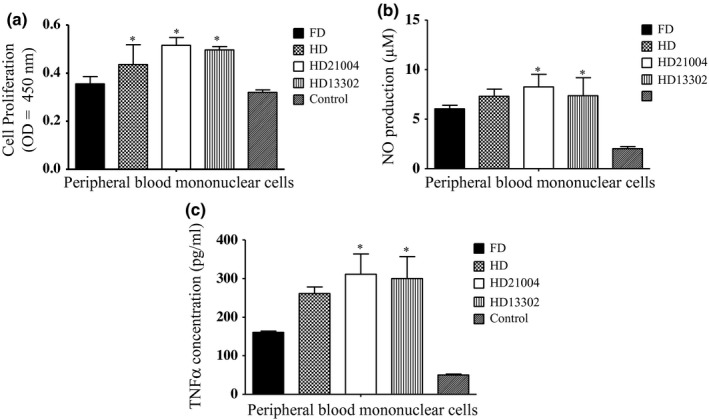
Effect of freeze‐dried garlic (FD), heat‐dried garlic (HD), heat‐dried garlic fermented with KCTC21004 (HD21004) and heat‐dried garlic fermented with KCTC13302 (HD13302) on (a) Cell proliferation, (b) NO production, and (c) TNF‐α production in peripheral blood mononuclear cells

Many studies have demonstrated that food samples with high antioxidant activity also have implications on immune stimulation (Ruiz‐Ruiz, Matus‐Basto, Acereto‐Escoffié, & Segura‐Campos, 2017; Zhang, Hu, Jiang, Zhao, & Zhu, 2018). For this reason, we ascertained the effect of the samples on NO and TNF‐α production in PBMCs. However, since some plant materials with good antioxidant activities may still be cytotoxic (Magalhães et al., 2010; Samia, Adam, Shigidi, & Hapke, 1998), we tested the ability of the samples to enhance PBMC proliferation (Figure 6). Generally, garlic treatment improved cell proliferation relative to untreated cells. However, all the heat‐dried fermented samples as well as the heat‐dried unfermented sample had similar cell proliferation enhancement abilities while freeze‐dried garlic had the lowest enhancement ability.

Nitric oxide acts as a defense molecule against bacteria and other pathogens. It also regulates the growth, activity, and death of many immune and inflammatory cell types (Randow, MacMicking, & James, 2013). Nitric oxide is easily oxidized to nitrite immediately after its generation and exists as nitrite both in intracellular and extracellular fluids. Thus, in this study, the content of nitrite, a stable product of NO, was measured to reflect the amount of NO as was reported by Green et al. (1982). It was observed that all the heat‐treated samples (HD, HD21004, and HD13302) induced larger quantities of NO which were far higher than the freeze‐dried garlic samples (*p* < 0.05). The levels of NO stimulated by the fermented samples were however not significantly different (*p* > 0.05). To confirm that proinflammatory cytokines were stimulated after NO production, we measured the levels of TNF‐α in the supernatants after the PBMCs were treated with the garlic extracts. All the garlic extracts induced more TNF‐α in the supernatants than the control samples (untreated cell). However, the levels of TNF‐α induced by HD21004 and HD13302 were not significantly different (*p* > 0.05) yet greater than that induced by HD. Our results agree with other studies that have reported a positive correlation between NO levels and proinflammatory cytokine production (Soufli, Toumi, Rafa, & Touil‐Boukoffa, 2016).

Since HD, HD21004, and HD13302 showed similar cell proliferation enhancement and had better NO and TNF‐α inducing ability than FD, we aimed to determine what compound might have been responsible for this observation. We chose to ascertain the amount of SAC in the samples since it is responsible for most of the multiple pharmacological effects of garlic (Kim et al., 2017). We observed that the amount of SAC in all the heat‐treated samples (whether fermented or unfermented) were about three times higher than the freeze‐dried garlic samples and this could account for the differences in the biological activities (Table 1). This observation supports an earlier report by Bae et al. (2014) who claimed that heat treatment of garlic could increase the levels of SAC compared to fresh garlic.

**Table 1:** *S*‐Allyl cysteine (SAC) analysis. The amount of SAC in the samples were determined and compared to the amount of SAC present in freeze‐dried raw garlic

Samples	Amount of SAC (mg/100 g)
FD	37.70 ± 0.47
HD	106.40 ± 0.49^a^
HD21004	116.50 ± 0.49
HD13302	108.83 ± 1.50^a^

*Notes*

The values represent means of three replicates ± *SD*.

Values with (^a^) are not significantly different (*p* > 0.05).

From this study, it has been demonstrated that drying garlic with heat could enhance the development of bioactive compounds which may be limited or absent in raw garlic samples (as seen in the significant differences in the activities of FD and HD). Subsequent solid‐state fermentation of the heat‐dried garlic with LAB could further improve the biological activities of the products but, this will depend on the bacterium used. Such a product could be useful as a functional food for boosting the immune system and promoting health.

## CONFLICT OF INTEREST

There are no conflicts of interests.

## ETHICAL STATEMENT

The current study was not required to complete an ethical assessment.

## References

[fsn3942-bib-0001] Ademiluyi, A. O. , & Oboh, G. (2011). Antioxidant properties of condiment produced from fermented bambara groundnut (*Vigna subterranea* L. Verdc). Journal of Food Biochemistry, 35(4), 1145–1160. 10.1111/j.1745-4514.2010.00441.x

[fsn3942-bib-0002] Adetuyi, F. O. , & Ibrahim, T. A. (2014). Effect of fermentation time on the phenolic, flavonoid and vitamin C contents and antioxidant activities of okra (*Abelmoschus esculentus*) seeds. Nigerian Food Journal, 32(2), 128–137. 10.1016/S0189-7241(15)30128-4

[fsn3942-bib-0003] Arranz, L. , Fernández, C. , Rodríguez, A. , Ribera, J. M. , & De Fuente, M. (2008). The glutathione precursor N‐acetylcysteine improves immune function in postmenopausal women. Free Radical Biology & Medicine, 45(9), 1252–1262. 10.1016/j.freeradbiomed.2008.07.014 18694818

[fsn3942-bib-0004] Bae, S. E. , Cho, S. Y. , Won, Y. D. , Lee, S. H. , & Park, H. (2014). Changes in S‐allyl cysteine contents and physicochemical properties of black garlic during heat treatment. LWT‐Food Science and Technology, 55(1), 397–402. 10.1016/j.lwt.2013.05.006

[fsn3942-bib-0005] Baluchnejadmojarad, T. , Kiasalari, Z. , Afshin‐Majd, S. , Ghasemi, Z. , & Roghani, M. (2017). S‐allyl cysteine ameliorates cognitive deficits in streptozotocin‐diabetic rats via suppression of oxidative stress, inflammation, and acetylcholinesterase. European Journal of Pharmacology, 794, 69–76. 10.1016/j.ejphar.2016.11.033 27887948

[fsn3942-bib-0006] Buelna‐Chontal, M. , & Zazueta, C. (2013). Redox activation of Nrf2 & NF‐*κ*B: A double end sword? Cellular Signalling, 25(12), 2548–2557. 10.1016/j.cellsig.2013.08.007 23993959

[fsn3942-bib-0007] Demir, N. , Yildiz, O. , Alpaslan, M. , & Hayaloglu, A. A. (2014). Evaluation of volatiles, phenolic compounds and antioxidant activities of rose hip (Rosa L.) fruits in Turkey. Lwt‐Food Science and Technology, 57(1), 126–133. 10.1016/j.lwt.2013.12.038

[fsn3942-bib-0008] García‐Villalón, A. L. , Amor, S. , Monge, L. , Fernández, N. , Prodanov, M. , Muñoz, M. , & Granado, M. (2016). In vitro studies of an aged black garlic extract enriched in S‐allylcysteine and polyphenols with cardioprotective effects. Journal of Functional Foods, 27, 189–200. 10.1016/j.jff.2016.08.062

[fsn3942-bib-0009] Green, L. C. , Wagner, D. A. , Glogowski, J. , Skipper, P. L. , Wishnok, J. S. , & Tannenbaum, S. R. (1982). Analysis of nitrate, nitrite, and [15N] nitrate in biological fluids. Analytical Biochemistry, 126(1), 131–138. 10.1016/0003-2697(82)90118-X 7181105

[fsn3942-bib-0010] Ham, J.‐S. , Lee, S.‐G. , Kim, M.‐K. , Oh, M.‐H. , Jeong, S.‐G. , Kim, D.‐H. , & Kang, D.‐K. (2010). Inhibitory activity of garlic fermented by *Pediococcus pentosaceus* KACC 91419 against antibiotic‐resistant pathogens. Asian‐Australasian Journal of Animal Sciences, 23(9), 1236–1243. 10.5713/ajas.2010.90457

[fsn3942-bib-0011] Ho, J.‐N. , Kang, M. , Lee, S. , Oh, J. J. , Hong, S. K. , Lee, S. E. , & Byun, S.‐S. (2018). Anticancer effect of S‐allyl‐L‐cysteine via induction of apoptosis in human bladder cancer cells. Oncology Letters, 15(1), 623–629. 29285203 10.3892/ol.2017.7280 PMC5738700

[fsn3942-bib-0012] Hosseini, S. E. , Bayat, M. , Shabani, S. , Mozaffari, N. , & Amir, S. (2014). Antibacterial effect of garlic aqueous extract on *Staphylococcus aureus* in Hamburger. Jundishapur Journal of Microbiology, 7(11), 1222–5. 10.5812/jjm.13134 PMC4332239 25774277

[fsn3942-bib-0013] Kim, S. , Park, S.‐L. , Lee, S. , Lee, S.‐Y. , Ko, S. , & Yoo, M. (2016). UPLC/ESI‐MS/MS analysis of compositional changes for organosulfur compounds in garlic (*Allium sativum* L.) during fermentation. Food Chemistry, 211, 555–559. 10.1016/j.foodchem.2016.05.102 27283666

[fsn3942-bib-0014] Kim, J. H. , Yu, S. H. , Cho, Y. J. , Pan, J. H. , Cho, H. T. , Kim, J. H. , & Jeong, Y. J. (2017). Preparation of S‐allylcysteine‐enriched black garlic juice and its antidiabetic effects in streptozotocin‐induced insulin‐deficient mice. Journal of Agricultural and Food Chemistry, 65(2), 358–363. 10.1021/acs.jafc.6b04948 28001066

[fsn3942-bib-0015] Kodera, Y. , Suzuki, A. , Imada, O. , Kasuga, S. , Sumioka, I. , Kanezawa, A. , & Masamoto, K. (2002). Physical, chemical, and biological properties of *S*‐allylcysteine, an amino acid derived from garlic. Journal of Agricultural and Food Chemistry, 50(3), 622–632. 10.1021/jf0106648 11804540

[fsn3942-bib-0016] Koley, T. K. , Kaur, C. , Nagal, S. , Walia, S. , & Jaggi, S. (2016). Antioxidant activity and phenolic content in genotypes of Indian jujube (*Zizyphus mauritiana* Lamk.). Arabian Journal of Chemistry, 9, S1044–S1052. 10.1016/j.arabjc.2011.11.005

[fsn3942-bib-0017] Lee, Y. G. , Cho, J.‐Y. , Kim, Y.‐M. , & Moon, J.‐H. (2016). Change in flavonoid composition and antioxidative activity during fermentation of onion (*Allium cepa* L.) by *Leuconostoc mesenteroides* with different salt concentrations. Journal of Food Science, 81(6), C1385–C1393. 10.1111/1750-3841.13329 27175820

[fsn3942-bib-0018] Lee, J.‐B. , Joo, W.‐H. , & Kwon, G.‐S. (2016). Biological activities of solid‐fermentation garlic with lactic acid bacteria. Journal of Life Science, 26(4), 446–452. 10.5352/JLS.2016.26.4.446

[fsn3942-bib-0019] Lee, H.‐S. , Lee, S.‐J. , Yu, H.‐J. , Lee, J.‐H. , & Cho, H.‐Y. (2017). Fermentation with Lactobacillus enhances the preventive effect of garlic extract on high fat diet‐induced hepatic steatosis in mice. Journal of Functional Foods, 30, 125–133. 10.1016/j.jff.2016.12.043

[fsn3942-bib-0020] Magalhães, H. , Paulo, M. P. , Moura, E. S. , Torres, M. R. , Alves, A. N. , Otília, D. L. , & Pessoa, C. (2010). In vitro and in vivo antiproliferative activity of *Calotropis procera* stem extracts. Anais da Academia Brasileira de Ciências, 82(2), 407–416. 10.1590/S0001-37652010000200017 20563422

[fsn3942-bib-0021] Nakajima, H. , Itakura, M. , Kubo, T. , Kaneshige, A. , Harada, N. , Izawa, T. , & Takeuchi, T. (2017). Glyceraldehyde‐3‐phosphate dehydrogenase (GAPDH) aggregation causes mitochondrial dysfunction during oxidative stress‐induced cell death. Journal of Biological Chemistry, M116, 759084. 10.1074/jbc.M116.759084 PMC5377786 28167533

[fsn3942-bib-0022] Randow, F. , MacMicking, J. D. , & James, L. C. (2013). Cellular self‐defense: How cell‐autonomous immunity protects against pathogens. Science, 340(6133), 701–706. 10.1126/science.1233028 23661752 PMC3863583

[fsn3942-bib-0023] Ried, K. (2016). Garlic lowers blood pressure in hypertensive individuals, regulates serum cholesterol, and stimulates immunity: An updated meta‐analysis and review. The Journal of Nutrition, 146(2), 389S–396S. 10.3945/jn.114.202192 26764326

[fsn3942-bib-0024] Ruiz‐Ruiz, J. C. , Matus‐Basto, A. J. , Acereto‐Escoffié, P. , & Segura‐Campos, M. R. (2017). Antioxidant and anti‐inflammatory activities of phenolic compounds isolated from *Melipona beecheii* honey. Food and Agricultural Immunology, 28(6), 1424–1437. 10.1080/09540105.2017.1347148

[fsn3942-bib-0025] Samia, M. A. , Adam, S. E. , Shigidi, M. T. , & Hapke, H. J. (1998). Studies on laticiferous plants: Toxic effects in goats of *Calotropis procera* latex given by different routes of administration. DTW. Deutsche tierarztliche Wochenschrift, 105(11), 425–427. 9857566

[fsn3942-bib-0026] Shen, C. Z. , Jun, H. Y. , Choi, S. H. , Kim, Y. M. , Jung, E. J. , Oh, G. S. , & Kim, I. K. (2010). Evaluation of antioxidant activities and active compounds separated from water soluble extracts of Korean black pine barks. Bulletin of the Korean Chemical Society, 31(12), 3567–3572. 10.5012/bkcs.2010.31.12.3567

[fsn3942-bib-0027] Shrestha, A. K. , Dahal, N. R. , & Ndungutse, V. (2010). Bacillus fermentation of soybean: A review. Journal of Food Science and Technology Nepal, 6, 1222–9.

[fsn3942-bib-0028] Soufli, I. , Toumi, R. , Rafa, H. , & Touil‐Boukoffa, C. (2016). Overview of cytokines and nitric oxide involvement in immuno‐pathogenesis of inflammatory bowel diseases. World Journal of Gastrointestinal Pharmacology and Therapeutics, 7(3), 353 10.4292/wjgpt.v7.i3.353 27602236 PMC4986402

[fsn3942-bib-0029] Sumioka, I. , Hayama, M. , Shimokawa, Y. , Shiraishi, S. , & Tokunaga, A. (2006). Lipid‐lowering effect of monascus garlic fermented extract (MGFE) in hyperlipidemic subjects. Hiroshima Journal of Medical Sciences, 55(2), 59. 16813070

[fsn3942-bib-0030] Wang, D. , Feng, Y. , Liu, J. , Yan, J. , Wang, M. , Sasaki, J. , & Lu, C. (2010). Black garlic (*Allium sativum*) extracts enhance the immune system. Medicinal and Aromatic Plant Science and Biotechnology, 4(1), 37–40.

[fsn3942-bib-0031] Wang, Y. , Zhang, J. , & Jing, H. (2016). Composition analysis of black garlics prepared by different garlic types of and processing technologies. Journal of Food Safety and Quality, 7(8), 3085–3091.

[fsn3942-bib-0032] Yang, E.‐J. , Kim, S.‐I. , Park, S.‐Y. , Bang, H.‐Y. , Jeong, J. H. , So, J.‐H. , & Song, K.‐S. (2012). Fermentation enhances the in vitro antioxidative effect of onion (*Allium cepa*) via an increase in quercetin content. Food and Chemical Toxicology, 50(6), 2042–2048. 10.1016/j.fct.2012.03.065 22504089

[fsn3942-bib-0033] Zhang, X.‐L. , Guo, Y.‐S. , Wang, C.‐H. , Li, G.‐Q. , Xu, J.‐J. , Chung, H. Y. , & Wang, G.‐C. (2014). Phenolic compounds from *Origanum vulgare* and their antioxidant and antiviral activities. Food Chemistry, 152, 300–306. 10.1016/j.foodchem.2013.11.153 24444941

[fsn3942-bib-0034] Zhang, T.‐T. , Hu, T. , Jiang, J.‐G. , Zhao, J.‐W. , & Zhu, W. (2018). Antioxidant and anti‐inflammatory effects of polyphenols extracted from *Ilex latifolia* Thunb. RSC Advances, 8(13), 7134–7141. 10.1039/C7RA13569F PMC9078438 35540363

[fsn3942-bib-0035] Zhang, W.‐F. , Xiong, Y.‐W. , Zhu, T.‐T. , Xiong, A.‐Z. , Bao, H.‐H. , & Cheng, X.‐S. (2017). MicroRNA let‐7 g inhibited hypoxia‐induced proliferation of PASMCs via G0/G1 cell cycle arrest by targeting c‐myc. Life Sciences, 170, 9–15. 10.1016/j.lfs.2016.11.020 27889560

[fsn3942-bib-0036] Zong, J. , & Martirosyan, D. M. (2018). Anticancer effects of garlic and garlic‐derived bioactive compounds and its potential status as functional food. Bioactive Compounds in Health and Disease, 1(2), 16–35.

